# Inducible knockdown of pregnancy‐associated plasma protein‐A gene expression in adult female mice extends life span

**DOI:** 10.1111/acel.12624

**Published:** 2017-06-09

**Authors:** Laurie K. Bale, Sally A. West, Cheryl A. Conover

**Affiliations:** ^1^ Endocrine Research Unit Mayo Clinic 200 First Street SW Rochester MN 55905 USA

**Keywords:** adult mice, inducible gene knockout, lifespan, mortality rates, pregnancy‐associated plasma protein‐A, tamoxifen

## Abstract

Pregnancy‐associated plasma protein‐A (PAPP‐A) knockout (KO) mice, generated through homologous recombination in embryonic stem cells, have a significantly increased lifespan compared to wild‐type littermates. However, it is unknown whether this longevity advantage would pertain to PAPP‐A gene deletion in adult animals. In the present study, we used tamoxifen (Tam)‐inducible Cre recombinase‐mediated excision of the floxed PAPP‐A (fPAPP‐A) gene in mice at 5 months of age. fPAPP‐A mice, which were either positive (pos) or negative (neg) for Tam‐Cre, received Tam treatment with quarterly boosters. Only female mice could be used with this experimental design. fPAPP‐A/neg and fPAPP‐A/pos mice had similar weights at the start of the experiment and showed equivalent weight gain. We found that fPAPP‐A/pos mice had a significant extension of life span (*P *=* *0.005). The median life span was increased by 21% for fPAPP‐A/pos compared to fPAPP‐A/neg mice. Analysis of mortality in life span quartiles indicated that the proportion of deaths of fPAPP‐A/pos mice were lower than fPAPP‐A/neg mice at young adult ages (*P *=* *0.002 for 601–800 days) and higher than fPAPP‐A/neg mice at older ages (*P *=* *0.004 for >1000 days). Thus, survival curves and age‐specific mortality indicate that female mice with knockdown of PAPP‐A gene expression as adults have an extended healthy life span.

Pregnancy‐associated plasma protein‐A (PAPP‐A) is the founding member of *pappalysins* in the metzincin superfamily of metalloproteinases (Boldt *et al*., 2001). Its only known function to date is to enhance local insulin‐like growth factor (IGF) availability for receptor activation through cleavage of inhibitory IGF binding proteins (Conover, 2012; Oxvig, 2015). As reduced IGF signaling has been shown to increase life span in a wide variety of species (Bartke, 2008), we postulated that loss of PAPP‐A would suppress IGF receptor signaling and extend life span. This was proven true in that both male and female PAPP‐A knockout (KO) mice lived significantly longer than their wild‐type littermates (Conover & Bale, 2007; Conover *et al*., 2010). The PAPP‐A KO mice were also resistant to the development of several age‐related diseases, such as atherosclerosis (Harrington *et al*., 2007). However, these mice were generated through homologous recombination in embryonic stem cells (Conover *et al*., 2004). To distinguish the impact of PAPP‐A deficiency in the adult from that during fetal and early postnatal development, we developed a mouse model suitable for tamoxifen (Tam)‐inducible, Cre recombinase‐mediated excision of the PAPP‐A gene (Conover *et al*., 2013a). In an atherosclerosis‐prone mouse model, Tam administration in adult mice inhibited established atherosclerotic plaque progression by 70% (Bale *et al*., 2014). In this study, we sought to answer the question of whether conditional reduction of PAPP‐A gene expression in adult mice would result in extended life span.

Female mice homozygous for floxed PAPP‐A (fPAPP‐A) and either positive (pos) or negative (neg) for Tam‐Cre were used in the life span study. Cre‐mediated excision and recombination were induced in five‐month‐old fPAPP‐A/pos mice with intraperitoneal (ip) injection of Tam (20 mg mL^−1^) in corn oil with 2% ethanol. fPAPP‐A/neg mice also received ip Tam as a control for any non‐specific effects of Tam treatment. Male mice could not be used in this life span study because Tam treatment can result in scrotal enlargement and subsequent complications, such as herniation (Reinert *et al*., 2012).The initial injection was with 6 mg of Tam/40 g body weight (bw) and then with 3 mg Tam/40 g bw weekly for 3 weeks. We had shown previously that this regimen produced maximum efficiency of excision in adult mice while limiting toxicity (Conover *et al*., 2013a). A 3 mg Tam/40 g bw ip booster was given every 4 months, thereafter, to induce excision and recombination in tissues that undergo considerable cell turnover. (Fig. S1, Supporting information, excision in the different tissues). Mice were examined daily throughout the study, including weekends and holidays. In spite of this monitoring, 17 fPAPP‐A/pos and 14 fPAPP‐A/neg mice were found dead in cage the next day. Mice were considered to be at end of life and euthanized by carbon dioxide inhalation, according to American Veterinary Medical Association Guidelines, if they were moribund and demonstrated one or more clinical signs suggesting imminent death: nonresponsive to being touched, labored breathing, failure to eat or drink. Mice euthanized because of ulcerative dermatitis unresponsive to treatment (five fPAPP‐A/neg; four fPAPP‐A/pos) were not included in the life span analyses. Inability to confirm genotypes at harvest also resulted in removal of mice from the analyses.

fPAPP‐A/neg and fPAPP‐A/pos mice had similar weights at the start of the experiment and showed equivalent weight gain up to 17 months of age Table S1 (Supporting Information). Survival distribution is presented in Fig. 1. We found that fPAPP‐A/pos mice had a significant extension of life span (*P *<* *0.005). The median life span was increased by 21% for fPAPP‐A/pos compared to fPAPP‐A/neg mice. Mortality in life span quartiles (Fig. 2) indicates that the proportion of deaths of fPAPP‐A/pos mice were lower than fPAPP‐A/neg mice at young adult ages (*P *=* *0.002 for 601–800 days) and higher than fPAPP‐A/neg mice at older ages (*P *=* *0.004 for >1000 days).

**Figure 1:**
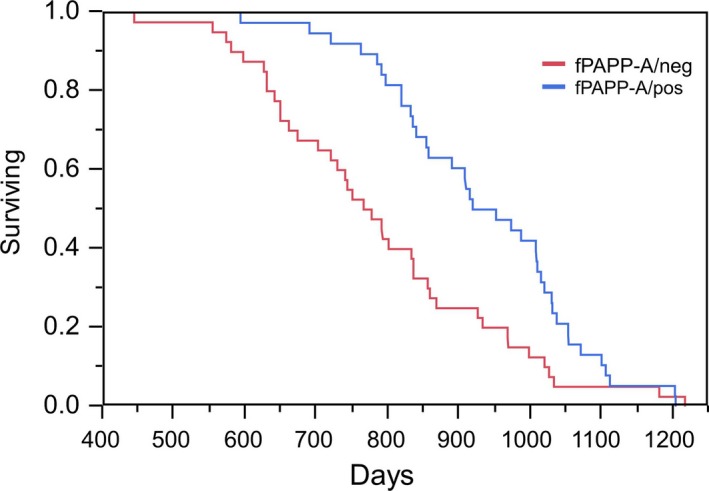
Survival distribution of fPAPP‐A/pos (blue line, *n* = 44) and fPAPP‐A/neg (red line, *n* = 40) mice. Kaplan–Meier curves were compared using log‐rank test. *P *=* *0.005.

**Figure 2:**
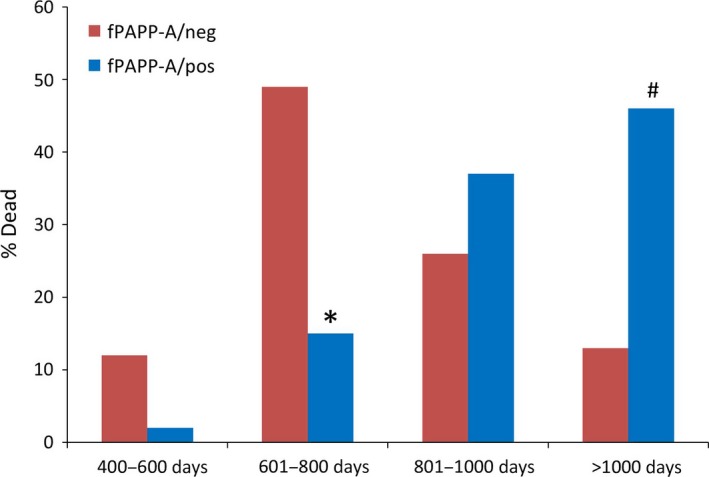
Age‐specific mortality. Life span quartiles of fPAPP‐A/pos (blue bars) and fPAPP‐A/neg (red bars) mice. Fisher's exact test was used to compare proportions of mice between groups.*P = 0.002, ^#^ *P* = 0.004

This study is the first to show that downregulation of PAPP‐A expression in adult mice can significantly extend life span. Importantly, this beneficial longevity phenotype is distinct from the dwarfism of long‐lived PAPP‐A KO, Ames dwarf, Snell dwarf and growth hormone receptor (GHR) KO mice with germ‐line mutations (Conover *et al*., 2004; Bartke, 2008). Thus, downregulation of PAPP‐A expression joins other treatment regimens, such as resveratrol, rapamycin and dietary restriction, which can extend life span when started in mice as adults (Weindruch & Walford, 1982; Baur *et al*., 2006; Harrison *et al*., 2009). In a recent study, inducible knockdown of the GHR in young adult female mice increased maximal, but not median, life span (Junilla *et al*., 2016). Tissue‐specific PAPP‐A KO models would provide insight into the tissues and organs that contribute to extended life span and healthspan.

An advantage of targeting PAPP‐A is the expectation of limited adverse side effects. We have already shown that global elimination of PAPP‐A gene expression has multiple positive effects, for example, resistance to: atherosclerotic plaque progression (Harrington *et al*., 2007), thymic involution and immune senescence (Vallejo *et al*., 2009), development of diabetic nephropathy (Mader *et al*., 2013), and visceral obesity (Conover *et al*., 2013b). One could also inhibit PAPP‐A's proteolytic activity to reduce IGF bioavailability for receptor activation. We have an immunoneutralizing monoclonal antibody specific for PAPP‐A (Mikkelsen *et al*., 2014), which has shown beneficial effects on ovarian cancer patient tumorgrafts in immunocompromised mice and on atherosclerotic plaque progression in apolipoprotein E‐deficient mice (Becker *et al*., 2015; Conover *et al*., 2016). Although these were studies performed in mice, there is growing evidence that PAPP‐A plays a role in atherosclerosis, diabetic nephropathy, visceral obesity, and several cancers in humans (Bayes‐Genis *et al*., 2001; Bulut *et al*., 2009; Conover, 2012; Huang *et al*., 2013; Mader *et al*., 2013; Henning *et al*., 2016). Thus, these data are foundational for pursuing small‐molecule inhibitors of PAPP‐A's proteolytic activity to promote healthy lifespan in humans.

## Funding

This work was supported by a grant from the National Institute on Aging (R01 AG028141 to CAC).

## Conflict of interest

The authors of this paper have no conflicts of interest to disclose.

## Author contributions

Laurie K. Bale and Sally A. West bred and genotyped the mice for inclusion in the study, administered treatments, monitored health of the mice on a daily basis, confirmed all genotypes at the end of the study, maintained database quality control, and performed the whole‐body fixation. They also contributed to the writing of the manuscript and reviewed and approved the final submission. Cheryl A. Conover had primary responsibility for experimental design, data analysis, and writing of the manuscript.

## Supporting information


**Fig. S1** Inducible PAPP‐A excision and recombination in various tissues.

**Table S1** Body weights of fPAPP‐A/neg and fPAPP‐A/pos mice.
