# Supplementary figures and images for: Inducible knockdown of pregnancy‐associated plasma protein‐A gene expression in adult female mice extends life span
Source: Aging Cell. 2017 Jun 9;16(4):895–7. doi: 10.1111/acel.12624 (PMC5506424; doi:10.1111/acel.12624)

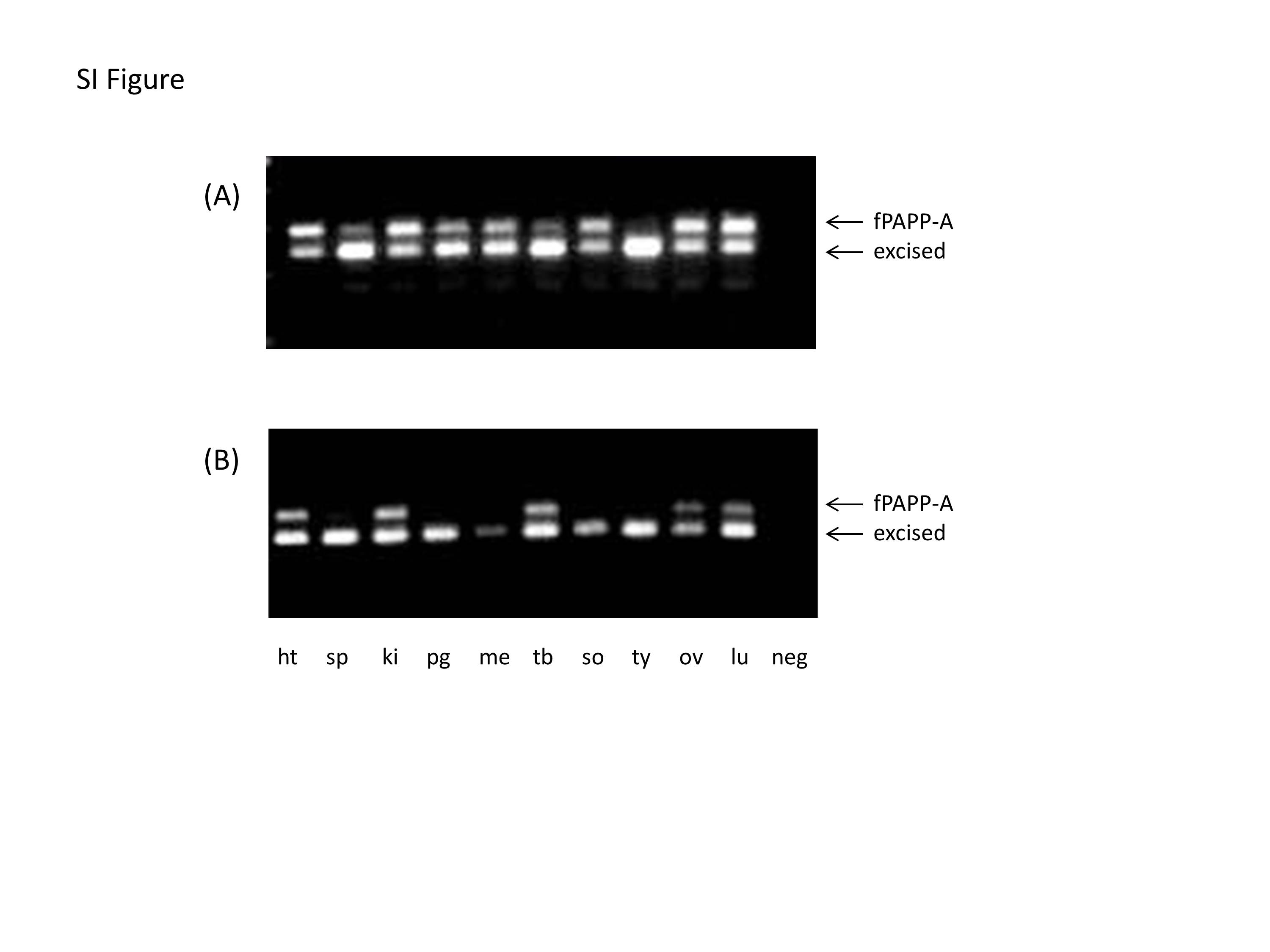

Supplement: Supplementary file 1 — Fig. S1 Inducible PAPP‐A excision and recombination in various tissues. [file ACEL-16-895-s001.jpg]
