# Supplementary material for: Inducible knockdown of pregnancy‐associated plasma protein‐A gene expression in adult female mice extends life span
Source: Aging Cell. 2017 Jun 9;16(4):895–7. doi: 10.1111/acel.12624 (PMC5506424; doi:10.1111/acel.12624)
Supplement: Supplementary file 2 — Table S1 Body weights of fPAPP‐A/neg and fPAPP‐A/pos mice. [file ACEL-16-895-s002.docx]

SI Table. Body weights of fPAPP-A/neg and fPAPP-A/pos mice

Weight (g)

| Months-of-age | fPAPP-A/neg | fPAPP-A/pos |
| --- | --- | --- |
| 5 | 25.7 +/- 0.54 | 26.1 +/- 0.52 |
| 9 | 28.7 +/- 0.63 | 28.6 +/- 0.69 |
| 13 | 30.1 +/- 0.66 | 30.2 +/- 0.72 |
| 17 | 30.7 +/- 0.70 | 30.3 +/- 0.78 |

Mice were weighed before the initial Tam injection at 5 months-of-age and boosters. The data (mean ± SEM, n = 54 fPAPP-A/neg, n = 53 fPAPP-A/pos) only go to 17 months, because after this time there was attrition in the numbers of mice, especially in the fPAPP-A/neg group.

SI Figure. Inducible PAPP-A excision and recombination in various tissues

PCRs of fPAPP-A excision (A) one week after the initial Tam treatment schedule in fPAPP-A/pos mice 5 months of age, and (B) in fPAPP-A/pos mice 35 months of age with seven additional Tam boosters. A 288-bp band represents intact fPAPP-A, and a 240-bp band represents the post-excision product.

ht, heart; sp, spleen; ki, kidney; pg, peri-gonadal fat; me, mesenteric fat; tb, tibia; so, soleus muscle; ty, thymus; ov; ovary; lu, lung; neg, negative control (H_2_O).
